# Written Informed Consent for Participation in a Study and Reduction in Consent Rate

**DOI:** 10.2188/jea.JE2008011

**Published:** 2008-12-17

**Authors:** Akiko Tamakoshi, Takashi Kawamura, Kenji Wakai, Masahiko Ando

**Affiliations:** 1 Department of Public Health, Aichi Medical University School of Medicine, Nagakute, Aichi, Japan; 2 Kyoto University Health Service, Kyoto, Japan; 3 Department of Preventive Medicine/Biostatistics and Medical Decision Making, Nagoya University Graduate School of Medicine, Nagoya, Japan

**Keywords:** Informed Consent, Consent Rate, Medical Check-up

## Abstract

**Background:**

The association between the method of obtaining informed consent and the consent rate in a cohort study, as well as the differences between consenters and non-consenters with regard to blood-sample donation are unclear.

**Methods:**

We measured the consent rates among 64-year-old residents who underwent medical checkups in a city for a cohort study consisting of a questionnaire survey and blood-sample donation and determined the influence of different approaches to informed consent and the participants’ characteristics on the consent rates.

**Results:**

Of 3,098 residents who underwent medical checkups over 10 years, 99.2% responded to the questionnaire survey, and 92.5% agreed to blood-sample donation. The consent rate for blood-sample donation after obtaining individual written informed consent was lower than that observed with the general-announcement approach. Differences in the consent rates for participation in the questionnaire study were, however, negligible. A higher percentage of men than women consented to donate blood samples. After adjustments for gender, it was observed that individuals with a history of hypertension and those without depression consented to blood-sample donation significantly more frequently.

**Conclusion:**

The consent rate for blood-sample donation to the study decreased when the opt-in approach with written consent was used. This decrease may introduce consent bias, and the method of obtaining informed consent should be revised.

## INTRODUCTION

In studies involving human subjects, it is desirable to obtain consent in order to adhere to the principle of respecting the patients’ intentions.^1^ However, the process of explaining the study contents and obtaining consent for participation in a study itself may introduce consent bias, and this complicates the generalization of the findings of epidemiological studies. Thus far, several studies^2^^-^^4^ have attempted to determine whether the method by which informed consent is obtained affects the consent rate in cohort studies. In contrast, response bias among Japanese people has not yet been assessed, except in a study by Matsui et al.^2^

From 1996 through 2005, we established a cohort of residents in a local community in central Japan. The approach to informed consent to a questionnaire survey and blood-sample donation changed during the study period. In the present study, we report the variations in the consent rates before and after the introduction of written informed consent as well as the consent rates for blood-sample donation according to participant characteristics.

## METHODS

The study cohort comprised people aged 64 years who participated in community-based comprehensive medical checkups; these people were followed up until they reached the age of 70. The cohort was designed with aim of clarifying the risk factors for lifestyle-related diseases. Since the people who were enrolled in the study in any given year would reach the age of 65 in that year, double registration did not occur over the study period. All participants were requested to fill out a self-administered questionnaire that focused on lifestyle habits and to donate blood samples when undergoing medical checkups. The questionnaire form was enclosed with the materials required for medical checkups and delivered to the participants in advance. The questionnaires were submitted at the site of medical checkup and checked for completeness. Blood samples for the study were collected through a single puncture during the medical checkup.

The outline of the study was depicted on the cover of the questionnaire. From 1996 through 2001, people who underwent medical checkup and submitted the questionnaires were considered to have consented to the questionnaire survey. In 2002, these participants were asked to check a designated box on the questionnaire. Since 2003, the consenters were asked to sign their name and write the date on a consent form. When possible, those who refused to participate in the questionnaire survey were asked to state their reasons by a trained staff member until 2001, but not since 2002.

From 1996 to 1999, public health nurses (PHNs) of the community explained the medical-checkup program to the subjects and then verbally requested that the subjects donate blood samples. Since 2000, an explanatory leaflet with an enclosed written consent form was delivered in advance to the participants along with the medical-checkup materials. Moreover, since the same year, at the beginning of medical checkups, one of the researchers instead of the PHNs provided general information about the study and requested the checkup participants to donate blood samples for the study. The participants submitted a written consent form before blood was drawn. Throughout the study period, a sheet that explained the blood-sample donation to the study was posted at the site of medical checkup, and inquiries about the study were welcomed by the attending researchers.

Since 2000, i.e., since the written consent form came into use, consent rates for blood-sample donation were assessed on the basis of participant characteristics, which were determined using information obtained from the questionnaires: gender, smoking habits, alcohol-drinking habits, sports activities, occupation, history of specific diseases, depression status, and activities of daily living (ADLs). The Geriatric Depression Scale^5^ was used to evaluate depression. The ADLs were compared using the Tokyo Metropolitan Institute of Gerontology (TMIG) index, which measures the capacity for activity in the elderly.^6^ The consent rate was compared using the χ^2^ test adjusted for gender by the Mantel-Haenszel method.

This study was approved by the ethics committee of Nagoya University School of Medicine in March 2002.

## RESULTS

Changes in the consent rates are shown in Table 1. From 1996 through 2001, 1,796 medical-checkup participants responded to the questionnaire survey, while 3 refused (consent rate, 99.8%). The only reason cited for refusal to participate was inconvenience in completing the questionnaire. When written consent was made mandatory for participation in the questionnaire survey in 2002, the consent rate decreased to 96.3-99.7% (mean 98.3%). In 2000, when the written-consent approach was first used for blood-sample donation, the consent rate dropped to 83.6-92.4% (mean 88.4%), but seemed to recover in the last year. Overall, 99.2% of the subjects participated in the questionnaire survey, and 92.5%, consented to blood-sample donation. The consent rates were consistently greater for the questionnaire survey than for blood-sample donation.

**Table 1.** Yearly change in the number of subjects and consenters.

		Consenters					

	Check-up participants	Questionnaire survey			Blood-sample donation		
	
Year		Approach	Number	Proportion (%)	Approach	Number	Proportion (%)
1996	253	GAA	253	100.0	GAA	253	100.0
1997	266		265	99.6		264	99.2
1998	268		268	100.0		268	100.0
1999	321		321	100.0		321	100.0
2000	324		323	99.7	WCA	289	89.2
2001	367		366	99.7		339	92.4
2002	334	WCA	327	97.9		306	91.6
2003	336		333	99.1		281	83.6
2004	297		286	96.3		251	84.5
2005	332		331	99.7		294	88.6

Total	3098		3073	99.2		2866	92.5

GAA: general-announcement approach

WCA: written-consent approach

Table 2 shows the consent rates for blood-sample donation according to the participants’ characteristics. The rate was slightly but significantly higher in the case of men than in the case of women (89.8% vs. 87.0%, *P* < 0.05). After adjustments for gender, the consent rates were significantly higher in those with a history of hypertension than in those without this history (91.9% vs. 87.3%, *P* < 0.01) and in those without depressive symptoms than in those with depressive symptoms (89.4% vs. 77.5%, *P* < 0.05). The consent rate was also higher, though not significantly so, in participants who were still at work at the time of the survey and in those with a history of cancer.

**Table 2.** Consent rates for blood-sample donation according to participant characteristics (2000-05).

	Questionnaire survey	Blood-sample donors	Consent rate(%)	
Gender				
Male	1023	919	89.8	
Female	967	841	87.0	*
Smoking habits				
Current smoker	335	302	90.1	
Quitter	578	522	90.3	
Never smoker	1077	936	86.9	
Drinking habits				
Current drinker	899	810	90.1	
Non-drinker/Quitter	1090	949	87.1	
Sports activities				
Regular	1033	929	89.9	
Not regular/None	911	803	88.1	
Work				
Yes	799	726	90.9	
No	1143	1003	87.8	#
Medical history of cancer				
Present	83	78	94.0	
Absent	1907	1682	88.2	#
Medical history of ischemic heart disease				
Present	25	20	80.0	
Absent	1965	1740	88.5	
Medical history of stroke				
Present	88	77	87.5	
Absent	1902	1683	88.5	
Medical history of diabetes				
Present	153	140	91.5	
Absent	1837	1620	88.2	
Medical history of hypertension				
Present	481	442	91.9	
Absent	1509	1318	87.3	**
Depression (Geriatric Depression Scale)				
Positive (≥11)	40	31	77.5	
Negative (≤10)	1913	1711	89.4	*
Activities of daily living (TMIG index)				
Lower (≤11)	446	396	88.8	
Higher (12 or 13)	1512	1349	89.2	

#: *P* < 0.1; *: *P* < 0.05; **: *P* < 0.01; adjusted for gender.

TMIG: Tokyo Metropolitan Institute of Gerontology

Subtotals do not add up to 1990 because some values are missing.

## DISCUSSION

This study showed that the consent rate for blood-sample donation to the study decreased after the introduction of the opt-in approach with the written-consent method, and some of the characteristics of the consenters differed from those of the non-consenters. The act of reading the explanatory leaflet at the checkup site or at home before the checkup might itself make participants more aware of the fact that they themselves or their blood samples would become the subjects of a study.

Junghans et al.^3^ showed a difference in the consent rates between the opt-in and opt-out methods of informed consent in observational studies. They conducted a randomized controlled trial on patient recruitment and compared the opt-in and opt-out approaches in a pilot investigation of an observational study. The recruitment rate was 38% in the opt-in arm and 50% in the opt-out arm. Matsui et al. compared the consent rates in 2 genetic subcohorts between participants who were provided extensive preliminary information and those who were not.^2^ The provision of extensive preliminary information reduced the participation rates and resulted in an odds ratio of 0.63-0.74. According to a review by Edwards et al.^7^ that focused on consent rates and the method of obtaining consent in clinical trials, the act of providing subjects more information and more time to arrive at a decision was associated with lower consent rates. These findings are consistent with our results in the present study. However, Edwards et al. indicated that high levels of knowledge were significantly associated with less anxiety in the study subjects, irrespective of the consent method, leading one to conclude the necessity of clear and readily understandable explanations.

The main feature of the present study is the internal comparison in a single community-based cohort, the participants of which consistently received preventive health services from the local government. This method minimizes possible confounding in the study. Nevertheless, some limitations must be acknowledged. During the 10-year observation period of the present study, opinions regarding epidemiological studies in Japan were revised. Public regulations (amendment to the Act on the National Basic Resident Register, 1999; Ethical Guidelines for Epidemiological Research,^8^ 2002; Act on the Protection of Personal Information, 2003) were established, and the mass media criticized several health-service studies that were conducted without obtaining appropriate informed consent from the research subjects.^9^ These changes might have been expected to decrease the consent rates in our study; however, few changes were observed during the period when the same approach to informed consent was used.

Dunn et al.^10^ compared consenters and non-consenters in 7 epidemiological studies in the UK, and found that men, youths, and people complaining of symptoms were more likely to consent to be subjects of a study. Another observational study found that men, elderly persons, and those with poor physical function tended to consent to home surveys and reviews of medical records.^11^ Moreover, in Japan, Matsui et al. reported that male sex and younger age were positive factors for participation in a genetic cohort study.^2^ In the present study, the consent rate was significantly higher for men, and those who were hypertensive and non-depressive. Although these findings have not been investigated, certain personalities might affect the willingness of people to participate in experimental studies. If consent rates vary widely with participants’ characteristics, it would be difficult to extrapolate the results of cohort studies obtained from the consenters only to the general population.

In conclusion, we found that the consent rate for blood-sample donation decreased with the introduction of the opt-in approach with the written-consent method, and consenters and non-consenters differed with regard to some characteristics. The method of obtaining informed consent may necessarily have to be revised to encourage people from all backgrounds to enroll in the study.

## References

[1] World Medical Association Declaration of Helsinki. Ethical Principles for Medical Research Involving Human Subjects. Available from: http://www.wma.net/e/policy/b3.htm (accessed November 8, 2007).

[2] Matsui K, Kita Y, Ueshima H. Informed consent, participation in, and withdrawal from a population based cohort study involving genetic analysis. J Med Ethics 2005;31:385-92. 10.1136/jme.2004.009530 15994356 PMC1734191

[3] Junghans C, Feder G, Hemingway H, Timmis A, Jones M. Recruiting patients to medical research: double blind randomized trial of “opt-in” versus “opt-out” strategies. BMJ 2005;331: 940. 10.1136/bmj.38583.625613.AE 16157604 PMC1261191

[4] Ronckers C, Land C, Hayes R, Verduijn P, van Leeuwen F. Factors impacting questionnaire response in a Dutch retrospective cohort study. Ann Epidemiol 2004;14:66-72. 10.1016/S1047-2797(03)00123-6 14664782

[5] Yesavage JA, Brink TL, Rose TL, Lum O, Huang V, Adey M, Development and validation of a geriatric depression screening scale: a preliminary report. J Psychiatr Res 1982-1983;17:37-49. 10.1016/0022-3956(82)90033-4 7183759

[6] Koyano W, Shibata H, Nakazato K, Haga H, Suyama Y Measurement of competence in the elderly living at home: Development of an index of competence. Jpn J Public Health 1987;34:109-14.

[7] Edwards S, Lilford R, Thornton J, Hewison J. Informed consent for clinical trials: In search of the “best” method. Soc Sci Med 1998;47:1825-40. 10.1016/S0277-9536(98)00235-4 9877351

[8] Ministry of Education, Culture, Sports, Science and Technology; Ministry of Health, Labour and Welfare. Ethical Guidelines for Epidemiological Research. Available from: http://www.niph.go.jp/english2/english%20ver/ethical-gl/guidelines.doc (accessed November 8, 2007).

[9] Nakayama T, Sakai M, Slingsby BT. Japan’s ethical guidelines for epidemiologic research: A history of their development. J Epidemiol 2005;15:107-12. 10.2188/jea.15.107 16141628 PMC7851070

[10] Dunn KM, Jordan K, Lacey RJ, Shapley M, Jinks C. Patterns of consent in epidemiologic research: Evidence from over 25,000 responders. Am J Epidemiol 2004;159:1087-94. 10.1093/aje/kwh141 15155293

[11] Buckley B, Murphy AW, Byrne M, Glynn L. Selection bias resulting from the requirement for prior consent in observational research: a community cohort of people with ischaemic heart disease. Heart 2007;93:1116-20. 10.1136/hrt.2006.111591 17502325 PMC1955022

